# The predictive role of symptoms in COVID-19 diagnostic models: A longitudinal insight

**DOI:** 10.1017/S0950268824000037

**Published:** 2024-01-22

**Authors:** Olivia Bird, Eva P. Galiza, David Neil Baxter, Marta Boffito, Duncan Browne, Fiona Burns, David R. Chadwick, Rebecca Clark, Catherine A. Cosgrove, James Galloway, Anna L. Goodman, Amardeep Heer, Andrew Higham, Shalini Iyengar, Christopher Jeanes, Philip A. Kalra, Christina Kyriakidou, Judy M. Bradley, Chigomezgo Munthali, Angela M. Minassian, Fiona McGill, Patrick Moore, Imrozia Munsoor, Helen Nicholls, Orod Osanlou, Jonathan Packham, Carol H. Pretswell, Alberto San Francisco Ramos, Dinesh Saralaya, Ray P. Sheridan, Richard Smith, Roy L. Soiza, Pauline A. Swift, Emma C. Thomson, Jeremy Turner, Marianne Elizabeth Viljoen, Paul T. Heath, Irina Chis Ster

**Affiliations:** 1 Vaccine Institute, St. George’s University of London, St. George’s University Hospitals National Health Service Foundation Trust, London, United Kingdom; 2 Oxford University Hospitals NHS Foundation Trust, Oxford, United Kingdom; 3 Medical Education, Stockport National Health Service Foundation Trust, Stepping Hill Hospital, Stockport, United Kingdom; 4 Chelsea and Westminster Hospital, National Health Service Foundation Trust, London, United Kingdom; 5 Faculty of Medicine, Imperial College London, London, United Kingdom; 6 Endocrinology/Diabetes/General Medicine, Royal Cornwall Hospitals National Health Service Trust, Truro, United Kingdom; 7 Faculty of Population Health Sciences, Institute for Global Health, University College London, and Royal Free London National Health Service Foundation Trust, London, United Kingdom; 8 Centre for Clinical Infection, South Tees Hospitals National Health Service Foundation Trust, James Cook University Hospital, Middlesbrough, United Kingdom; 9 Layton Medical Centre, Blackpool, United Kingdom; 10 Centre for Rheumatic Disease, Kings College London, London, United Kingdom; 11 Department of Infectious Diseases, Guy’s and St Thomas’ National Health Service Foundation Trust, London, United Kingdom; 12 Medical Research Council Clinical Trials Unit, University College London, London, United Kingdom; 13 Lakeside Healthcare Research, Lakeside Surgeries Corby, Northants, United Kingdom; 14 Gastrointestinal and Liver Services, University Hospitals of Morecambe Bay National Health Service Foundation Trust, Kendal, United Kingdom; 15 Accelerated Enrollment Solutions, Synexus Hexham Dedicated Research Site, Hexham General Hospital, Hexham, United Kingdom; 16 Department of Microbiology, Norfolk and Norwich University Hospitals National Health Service Foundation Trust, Norfolk, United Kingdom; 17 Nephrology, Salford Royal Hospital, Northern Care Alliance National Health Service Foundation Trust, Salford, United Kingdom; 18 Accelerated Enrollment Solutions, Synexus Midlands Dedicated Research Site, Birmingham, United Kingdom; 19 Dentistry and Biomedical Sciences, School of Medicine, Wellcome-Wolfson Institute for Experimental Medicine, Queen’s University of Belfast, Belfast, United Kingdom; 20 Accelerated Enrollment Solutions, Synexus Merseyside Dedicated Research Site, Burlington House, Liverpool, United Kingdom; 21 Centre for Clinical Vaccinology and Tropical Medicine, University of Oxford, Oxford, United Kingdom; 22 Oxford Health National Health Service Foundation Trust, Warneford Hospital, Oxford, United Kingdom; 23 Department of Microbiology, Leeds Teaching Hospitals National Health Service Trust, Leeds, United Kingdom; 24 The Adam Practice, Dorset, United Kingdom; 25 University Hospital Southampton National Health Service Foundation Trust, Southampton, United Kingdom; 26 Accelerated Enrollment Solutions, Synexus Glasgow Dedicated Research Site, Glasgow, United Kingdom; 27 Accelerated Enrollment Solutions, Synexus Wales Dedicated Research Site, Cardiff, United Kingdom; 28 School of Medical Sciences (Pharmacology/Pharmacy), Bangor University, Wales, United Kingdom; 29 Clinical Pharmacology and Therapeutics/General Internal Medicine, Betsi Cadwaladr University Health Board, Wales, United Kingdom; 30 Academic Unit of Population and Lifespan Sciences, University of Nottingham, Nottingham, United Kingdom; 31 Department of Rheumatology, Haywood Hospital, Midlands Partnership National Health Service Foundation Trust, Stafford, United Kingdom; 32 Accelerated Enrollment Solutions, Synexus Lancashire Dedicated Research Site, Matrix Park Buckshaw Village, Chorley, United Kingdom; 33 National Institute for Health Research, Patient Recruitment Centre, Bradford Teaching Hospitals National Health Service Foundation Trust, Bradford, United Kingdom; 34 Geriatric Medicine, Royal Devon University Healthcare, Exeter, United Kingdom; 35 Department of Nephrology, East Suffolk and North Essex National Health Service Foundation Trust, Colchester, United Kingdom; 36 Aberdeen Royal Infirmary and Ageing Clinical and Experimental Research Group, University of Aberdeen, Aberdeen, United Kingdom; 37 Renal Services, Epsom and St Helier University Hospitals National Health Service Trust, London, United Kingdom; 38 School of Infection & Immunity, Medical Research Council-University of Glasgow Centre for Virus Research, and Queen Elizabeth University Hospital, National Health Service Greater Glasgow & Clyde, Glasgow, United Kingdom; 39 Department of Diabetes and Endocrinology, Norfolk and Norwich University Hospitals National Health Service Foundation Trust, Norfolk, United Kingdom; 40 Accelerated Enrollment Solutions, Synexus Manchester Dedicated Research Site, Kilburn House, Manchester, United Kingdom; 41 Institute of Infection and Immunity, George’s University of London, London, United Kingdom

**Keywords:** coronavirus, longitudinal data, symptoms dynamics

## Abstract

To investigate the symptoms of SARS-CoV-2 infection, their dynamics and their discriminatory power for the disease using longitudinally, prospectively collected information reported at the time of their occurrence. We have analysed data from a large phase 3 clinical UK COVID-19 vaccine trial. The alpha variant was the predominant strain. Participants were assessed for SARS-CoV-2 infection via nasal/throat PCR at recruitment, vaccination appointments, and when symptomatic. Statistical techniques were implemented to infer estimates representative of the UK population, accounting for multiple symptomatic episodes associated with one individual. An optimal diagnostic model for SARS-CoV-2 infection was derived. The 4-month prevalence of SARS-CoV-2 was 2.1%; increasing to 19.4% (16.0%–22.7%) in participants reporting loss of appetite and 31.9% (27.1%–36.8%) in those with anosmia/ageusia. The model identified anosmia and/or ageusia, fever, congestion, and cough to be significantly associated with SARS-CoV-2 infection. Symptoms’ dynamics were vastly different in the two groups; after a slow start peaking later and lasting longer in PCR+ participants, whilst exhibiting a consistent decline in PCR- participants, with, on average, fewer than 3 days of symptoms reported. Anosmia/ageusia peaked late in confirmed SARS-CoV-2 infection (day 12), indicating a low discrimination power for early disease diagnosis.

## Introduction

The SARS-COV-2 pandemic has contributed to significant global morbidity and mortality. As of March 7, 2023, there have been over 759 million cases of COVID-19, including 6.8 million deaths [1]. The burden of disease was greatly felt by all public health organizations, particularly by healthcare systems that were frequently put under strain as they managed surges of infections [2]. The unprecedented scale and speed of the pandemic, its similarities to influenza, and the three major foci of care homes, hospitals, and the community, proved to be a challenging combination for devising a standard list of symptoms for COVID-19. Accurate recognition of the symptoms that indicated infection and warranted urgent testing was particularly important in the early stages of the pandemic when polymerase chain reaction (PCR) test kits were in demand [3].

The gold standard for diagnosing SARS-COV-2 infection is an oropharyngeal/nasal PCR swab, although latterly lateral flow tests are used for rapid diagnosis [4]. In the UK, PCR testing was initially prioritized for those presenting with a new (or worsening) cough, fever, or breathlessness [5]. However other symptoms such as altered or loss of smell (anosmia) or taste (ageusia), and gastrointestinal symptoms (such as loss of appetite and diarrhoea) have also been associated with COVID-19 [6–8]. In a Cochrane Review (2021), mainly based on more severely affected populations (e.g. hospitalized patients), the pooled specificities for anosmia and ageusia were high (90.5%), suggesting these symptoms may be a useful marker for COVID-19 [9]. The updated review (2022) concluded that most other individual symptoms had poor diagnostic accuracy [10].

In a study of 483 subjects in Washington D.C., 42% of whom were healthcare or essential workers, aged between 25 and 44 years, who retrospectively reported symptoms, 27% were reported to be PCR positive. Wojtusiak et al. concluded that clusters of symptoms are more predictive of COVID-19 than any one specific symptom [11]. In a different study, the same authors also examined the importance of the order of symptom occurrence in deriving a disease diagnostic model [12]. A meta-analysis based on sample data collected from nine established longitudinal cohorts designed a four-category cross-sectional outcome aiming to capture characteristics of long COVID in the UK population [13]. Based on questionnaires completed by subsets of participants between July 2020 and September 2021 and self-reported COVID results as well as the presence/absence of symptoms, the meta-analysis demonstrated considerable heterogeneity between studies [13].

The observation of previous research shows that there is a great deal of variation in data collection methods (e.g. smartphone apps, patient records [14–16]), epidemiological heterogeneity of study populations (e.g. hospitals, intensive care units, care homes [13–15]), and different reporting methods (e.g. self-reports, interviews [17]). As symptoms develop over time, cross-sectional outcomes and retrospectively collected information on symptoms may be difficult to relate to COVID-19 onset which is also known to have a variable incubation period (2–14 days) [18]. The Zoe Health Study compared three different symptom-based diagnostic models for SARS-CoV-2 and investigated the effect of demographic variables on the models’ performance metrics and found that the discrimination power of all models improved with the number of days of symptoms included, whilst the most relevant symptoms for detecting COVID-19 were anosmia and chest pain [12].

The phase 3 Novavax COVID-19 clinical trial in the UK was conducted at 33 sites and recruited 15,185 participants [19]. Its primary aim was to evaluate the efficacy and safety of the vaccine. We used the prospectively reported symptoms of possible SARS-CoV-2 infection to assess the discrimination power of individual symptoms and to investigate an optimal combination to generate a diagnostic model for the presence of SARS-CoV-2 infection in the UK population.

## Methods

The data for this analysis were provided by Novavax, Inc. [19]. The methods and results of the trial are described elsewhere [19]. Data included are from October 28, 2020 to February 28, 2021.

### Monitoring for COVID-19

All participants had a SARS-CoV-2 PCR test performed at recruitment and were tested for symptomatic infections throughout the study. Participants were instructed to contact the study team within 24 h if they self-assessed COVID-19 symptoms (Table 1), triggering a surveillance visit. Throat/nasal swabs were self-collected by participants approximately 24 h after the onset of symptoms, then daily for up to 3 days. A participant with suspected or confirmed COVID-19 was asked to complete a symptom diary, starting on their first day of symptoms, reporting daily for a minimum of 10 days (even if their symptoms resolved and regardless of SARS-CoV-2 PCR result). Participants with confirmed symptomatic COVID-19, signified by a positive PCR test, continued documenting their symptoms until resolution. Virologic confirmation was performed by PCR assay at the U.K. Department of Health and Social Care laboratories with the TaqPath system (Thermo Fisher Scientific).

**Table 1.** Qualifying symptoms of suspected COVID-19

Fever (referred to as ‘fever’) New onset cough (referred to as ‘cough’) New onset or worsening of shortness of breath or difficulty breathing compared to recruitment time (referred to as ‘breathlessness’) New onset fatigue (referred to as ‘fatigue’) New onset generalized muscle or body aches (referred to as ‘myalgia’) New onset headache (referred to as ‘headaches’) New loss of taste or smell (referred to as ‘loss of taste/smell’) New loss of appetite (referred to as ‘anorexia’) Acute onset of sore throat (referred to as ‘sore throat’) Acute onset congestion (referred to as ‘congestion’) Acute onset runny nose (referred to as ‘runny nose’) New onset of chills (referred to as ‘chills’) New onset of nausea (referred to as ‘nausea’) New onset of diarrhoea (referred to as ‘diarrhoea’)

### Statistical methodology

The main objective was to construct an optimal diagnostic model for COVID-19 based on participants’ symptoms and to highlight differences in the dynamics of specific symptoms in groups defined by participants who experienced COVID-19 and those who did not. To extrapolate the results to the UK population, we started by plotting and empirically comparing the distribution of age, gender, and ethnicity distributions in the sample data to that of the UK population [20–22]. We then used post-stratification techniques for incorporating population demographic distributions [23]. This procedure allowed us to produce estimates generalizable to the UK community population. Weights were derived and assigned to each participant such that the subsequent estimation procedures inflated the effect of under-represented groups (e.g. young ethnic minorities) and depressed the effect of overrepresented groups in the sample (e.g. old whites).

We constructed a master file that included multiple PCR tests per participant and multiple symptomatic episodes. The resulting data have a hierarchical structure with implications on the subsequent choice of analyses and estimation procedures (details in the Supplementary material). Participants were initially grouped by their PCR results, that is, participants with at least one PCR positive result (PCR+) and those always negative (PCR-). We reported the frequency and proportion of the symptomatic participants in the two groups. We estimated the probabilities of testing positive given a specific symptomatic episode and the mean number of reports (or number of days) of a specific symptom within an illness episode. We also investigated the symptom report dynamics and explored the extent to which symptoms were associated with demographics. These analyses identified the main confounder candidates and their potential influence on the subsequent receiver operating characteristic (ROC) analyses.

Non-parametric techniques such as local polynomial smoothing have been used to fit curves on the daily probabilities of the reports in the PCR+ and PCR− participants. A heatmap of daily probabilities of reported symptoms has also been presented in ascending order of their magnitude on the first day in positive patients.

We assessed the effect of reporting the number of days of each specific symptom on the probability of testing PCR+ vs. PCR−, measured as odds ratios and their 95%CIs. We derived a symptom-based diagnostic model using two-level logistic regression and evaluated the discriminatory power of this model using the area under the curve (AUC) as a metric for its discrimination. We also performed a two-stage process ROC analysis [24]. The technique allows multiple episodes to be associated with an individual, and adjustments using population weights. The result is an estimate of the ROC curve for each specific symptom as a function of age and ethnicity – known as a covariate-specific ROC curve [24]. Using these techniques, we also highlighted the increasing discrimination power of individual symptoms based on the temporally ordered reports restricted to the first 1, 2, 3 to longer than 15 days after the start of the symptomatic illness episode. The effect of age and ethnicity on the discrimination power of individual symptoms was also evaluated. More details are in the Supplementary material.

## Results

### Data summary

**Figure 1.**
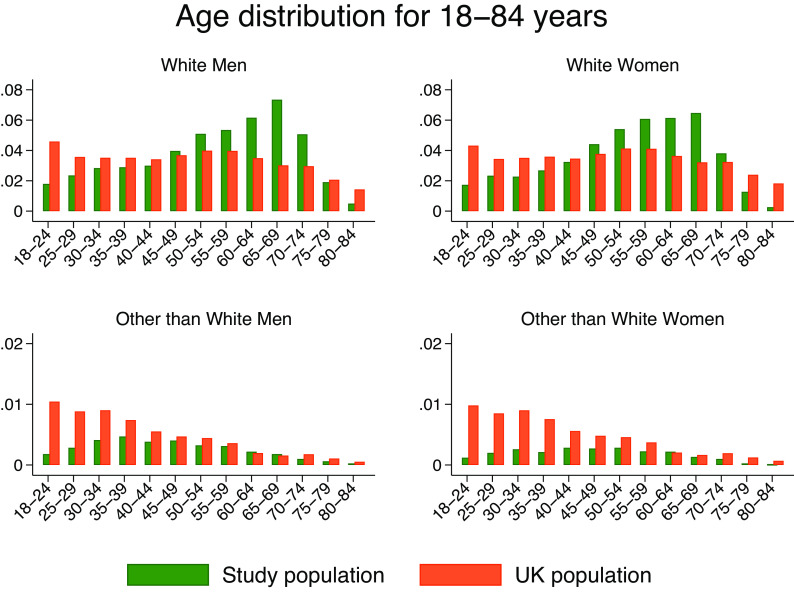
Age distribution in the study sample compared to that of the UK population, stratified by gender and ethnicity.

Table 2 shows a simplified picture of the data based on a binary assessment. Of 15,139 participants, 317 (2.1%) had a PCR+ episode and 3,320 (21.9%) had at least one symptomatic episode. 8% (266/3320) of the symptomatic population were PCR+ and 84% (266/317) of the PCR+ participants reported symptoms. Figure 1 shows the age distribution against that of the UK population stratified by gender and ethnicity [20–22]. These data have been used to calculate the weights associated with our analyses.

**Table 2.** PCR and symptomatic status of all study participants; 3,320 (21.9%) of all participants had at least one symptomatic episode and 317 (2.1%) of all had a PCR+ episode

	Overall		
	PCR−	PCR+	Total
No symptomatic episode	11,768	51	11,819
At least one symptomatic episode	3,054	266	3,320 (21.9%)
	14,822	317 (2.1%)	15,139

Table 3 presents demographic data stratified by PCR status. The comorbidities variable indicates the presence of at least one comorbidity. COVID-19 was directly associated with younger age, that is, 1 year increase in age decreased the OR of COVID-19 by a small yet significant factor of 0.98 (*p* < 0.001). Ethnic minorities (excluding white) were twice as likely to test positive than their white counterparts, that is, OR = 1.924 (95%CI (1.169, 3.167)). The other than white category included Asians (*n* = 462 (3.1%)), Black (*n* = 60 (0.4%)), and others (*n* = 153 (1%)).

**Table 3.** Cohort demographic characteristics stratified by participant PCR status

Variable	Summary/category	All	PCR+	PCR−	OR	*P*-value	95%CI low	95%CI high
		**15,139**	**317**	**14,822**				
Age	Mean/SD	53.1/14.9	49.2/13.6	53.2/14.9	0.983	**<0.001**	0.975	0.991
(years)	Median (IQR)	55(42, 65)	51(38, 60)	55(43, 65)				
	Min–max	18–84	18–79	18–84				
Gender	Male	7,808(51.6%)	152(48.0%)	7,656(51.6%)	1.086	0.550	.829	1.423
	Female	7,331(48.4%)	165(52.1%)	7,166(48.4%)				
Ethnicity	White	14,280 (94.3%)	288(90.9%)	13,992(94.4%)	1.924	**0.010**	1.169	3.167
	BAME	675(4.5%)	26(8.2%)	649(4.4%)				
	Missing	184 (1.2%)	3(0.95%)	181(1.2%)				
BMI	Mean/SD	27.6/5.3	28.2/5.6	27.6/5.3	1.003	0.845	.976	1.030
	Median (IQR)	26.7(23.9–30.4)	27.1(24.1–31.6)	26.7(23.9–30.4)				
	Min–max	15.1–55	16.8–53	15.1–55				
	Missing	412(2.7)	7(2.5)	405(2.7)				
BMI > 30	No	10,777(71.2%)	216(68.1%)	10,561(71.3%)	1.002	0.991	.759	1.321
	Yes	3,950(26.1%)	94(29.7%)	3,856(26.0%)				
	Missing	412(2.7%)	7(2.2%)	405(2.7%)				
Presence of comorbidities	No	8,372 (55.3%)	177(55.8%)	8,195(55.3%)	0.816	0.128	.628	1.060
	Yes	6,767 (44.7%)	140 (44.2%)	6,627(44.7%)				

The ORs measure univariate associations between the PCR status and population characteristics, irrespective of the presence of symptoms. Statistically significant associations are marked in bold.

**Figure 2.**
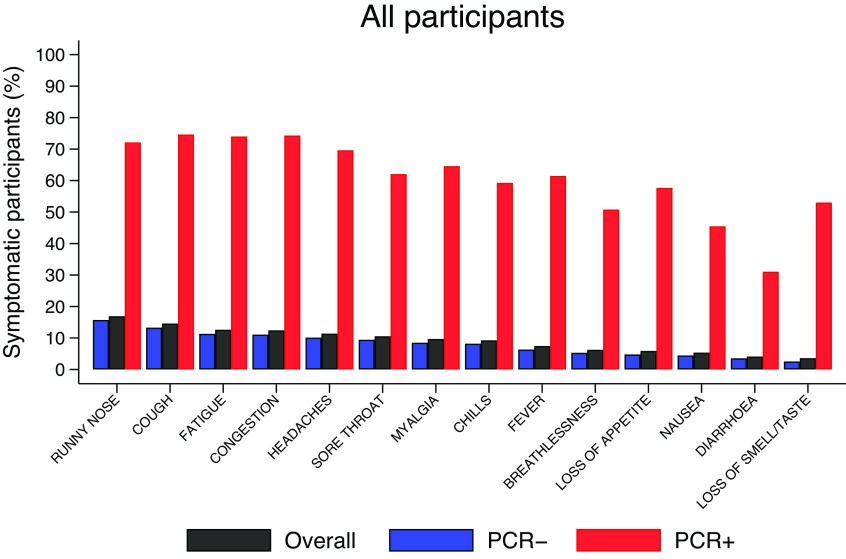
Proportions of participants with specific symptoms, overall, and stratified by PCR status, as shown in the Supplementary Material. For example, overall, 16.9% of all participants reported runny nose at least once but the figure is much higher (72.6%) among PCR+ contrasting with 15.7% among PCR−.

Summary symptoms data (overall and stratified by PCR status) are presented in the Supplementary Material and illustrated in Figure 2. Runny nose (16.9%) was the most reported symptom in this cohort, followed by cough (14.6%) and tiredness (12.6%). Nausea (5.3%), diarrhoea (4.1%), and anosmia/ageusia (3.6%) were the least reported. This ordering is preserved in PCR− participants; however, in PCR+ participants cough (75.1%) was the most frequent symptom, followed by congestion (74.8%) and tiredness (74.4%). Anosmia/ageusia was reported by 53.3% of PCR+ participants versus 2.5% of PCR− participants.

### The probabilities of PCR status by specific symptom reports

**Figure 3.**
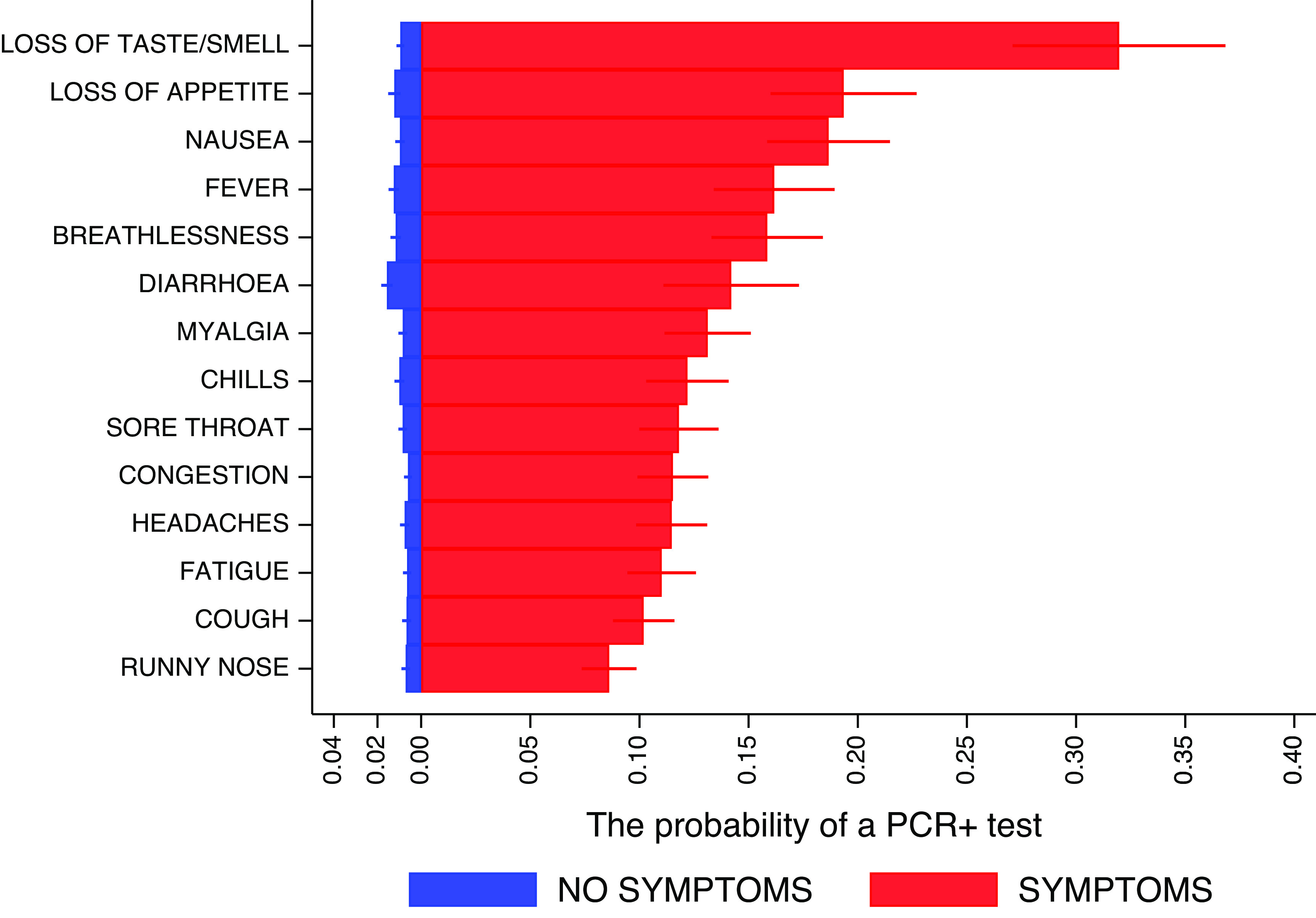
Predicted probabilities of PCR+ status, stratified by the presence of specific symptoms, and their 95%CIs. Predictions related to each specific symptom are unadjusted for the others and are based on a binary regression with robust standard errors accounting for multiple episodes with events associated with a participant. For example, in participants with loss of taste or smell, regardless of the presence or absence of other symptoms, the probability of a positive PCR test is 0.319 (31.9%).

Figure 3 shows the probabilities of testing PCR+ conditioned on each symptom (reported at least once). The prevalence of COVID-19 was 31.9% (27.1%–36.8%) in those reporting anosmia/ageusia and 19.4% (16%–22.7%) for loss of appetite.

### The number of specific symptom analyses

**Figure 4.**
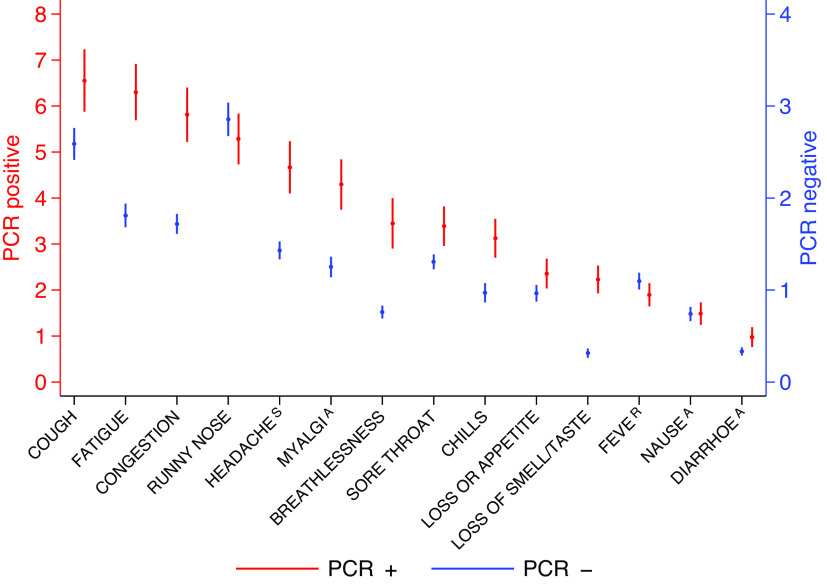
Predicted mean of number of days specific symptoms were reported during an episode and their 95%CIs. The red values (PCR+) are referred to the left axis and the blue values (PCR−) are referred to the right axis. The analysis is restricted to symptomatic participants only. For example, for those participants reporting cough as part of an episode, the mean of the number of days was 6–7 days in PCR+ participants and 2–3 days in PCR−.

Figure 4 shows the mean number of days (and their 95%CIs) that each specific symptom was reported during a symptomatic episode, stratified by PCR status. PCR+ participants reported a significantly longer duration of specific symptoms compared to PCR− participants. For example, the mean number of days of cough was 6–7 in PCR+ participants and 2–3 in PCR− participants.

Table 4 presents an exploratory analysis on the rate ratios (fold-effects) as measures of associations between the mean number of days of specific symptoms with population characteristics, this has been also analyzed in the PCR+ subgroup in the Supplementary Material. From Table 4, we learn that age was directly associated with an increased number of reports of runny nose, cough, and loss of appetite, but inversely associated with sore throat and anosmia/ageusia. Women reported 24.3% (95%CI (11.4%, 38.7%)) more headaches than men. Other than white participants reported fewer symptoms than white participants; for runny nose by a factor of 0.76 (95%CI (0.65, 0.89)), cough (by a factor of 0.77 (95% (0.62, 0.95)), and congestion (by a factor of 0.77 (95% (0.62, 0.96)). Increasing BMI was associated with increased reporting of myalgia (*P* = 0.033) and breathlessness (*p* < 0.001). Those with co-morbidities reported 18.5% (95%CI (8.1%, 29.8%)) more days of cough, 16.1% (95%CI (1.9%, 32.2%)) more days of myalgia, and 22.4% (95%CI (3.6%, 44.5%)) more days of breathlessness on average, than those without co-morbidities (Table 4).

**Table 4.** Fold-effects (risk ratios) of demographics and their 95%CIs on the mean number of days of specific symptoms reported during a symptomatic episode

	Age				Gender				Ethnicity				BMI				Comorbidities			
	RR	*P*-value	95%CI low–high		RR	*P*-value	95%CIlow–high		RR	*P*-value	95%CI low–high		RR	*P*-value	95%CI low–high		RR	*P*-value	95%CIlow–high	
Runny nose	1.005	**<0.001**	1.003	1.008	0.960	0.301	0.888	1.037	0.758	**0.001**	0.645	0.891	0.999	0.765	0.992	1.006	1.076	0.060	0.997	1.162
Cough	1.006	**<0.001**	1.003	1.009	0.966	0.477	0.879	1.062	0.769	**0.014**	0.624	0.949	1.005	0.140	0.998	1.013	1.185	**<0.001**	1.081	1.298
Fatigue	0.998	0.307	0.994	1.002	1.040	0.447	0.940	1.151	0.885	0.210	0.731	1.071	1.004	0.315	0.996	1.012	1.081	0.118	0.980	1.192
Congestion	0.997	0.182	0.994	1.001	1.049	0.370	0.945	1.164	0.773	**0.020**	0.622	0.961	1.005	0.214	0.997	1.014	1.050	0.352	0.948	1.162
Headaches	0.998	0.265	0.994	1.002	1.243	**<0.001**	1.114	1.387	0.894	0.386	0.694	1.152	1.005	0.329	0.995	1.014	1.047	0.408	0.939	1.167
Sore throat	0.995	**0.021**	0.990	0.999	1.056	0.419	0.925	1.205	0.887	0.488	0.633	1.244	0.998	0.781	0.988	1.009	1.039	0.567	0.912	1.182
Myalgia	1.005	0.060	1.000	1.010	0.975	0.713	0.853	1.115	0.820	0.101	0.647	1.040	1.012	**0.033**	1.001	1.023	1.161	**0.025**	1.019	1.322
Chills	1.001	0.786	0.994	1.008	1.043	0.661	0.865	1.257	0.751	0.080	0.545	1.035	1.003	0.718	0.989	1.017	1.102	0.282	0.923	1.315
Fever	0.999	0.842	0.994	1.005	1.052	0.573	0.881	1.257	0.852	0.453	0.561	1.295	1.014	0.033	1.001	1.027	1.111	0.227	0.936	1.319
Breathlessness	1.001	0.740	0.995	1.007	1.043	0.606	0.888	1.226	0.684	0.059	0.461	1.014	1.024	**<0.001**	1.012	1.037	1.224	**0.017**	1.036	1.445
Anorexia	1.009	**0.018**	1.001	1.016	1.021	0.822	0.849	1.228	0.720	0.139	0.466	1.113	1.012	0.066	0.999	1.025	1.184	0.070	0.986	1.420
Nausea	1.002	0.499	0.995	1.009	1.106	0.375	0.885	1.382	0.759	0.309	0.446	1.291	1.008	0.273	0.994	1.023	1.061	0.588	0.856	1.317
Diarrhoea	0.997	0.494	0.990	1.005	0.900	0.426	0.696	1.166	1.168	0.475	0.763	1.787	1.008	0.416	0.989	1.026	1.137	0.328	0.879	1.471
Loss of smell/taste	0.989	**0.018**	0.979	0.998	1.239	0.112	0.951	1.614	0.894	0.763	0.433	1.847	0.994	0.656	0.966	1.022	0.802	0.105	0.614	1.047

The estimation uses a Poisson zero-inflated model on the number of reports of an episode and allows for multiple episodes with events associated with one participant. Statistically significant associations are marked in bold.

In those with a positive PCR (Supplementary Material), many of these trends remained significant, for example, the effect of age on myalgia (*P* = 0.039) and loss of appetite (*P* = 0.012), the effect of gender on headaches (*P* = 0.033), of ethnicity on congestion (*P* = 0.002) and of BMI on breathlessness (*P* = 0.012). Increased BMI was associated with longer duration of cough (*P* = 0.022).

**Figure 5.**
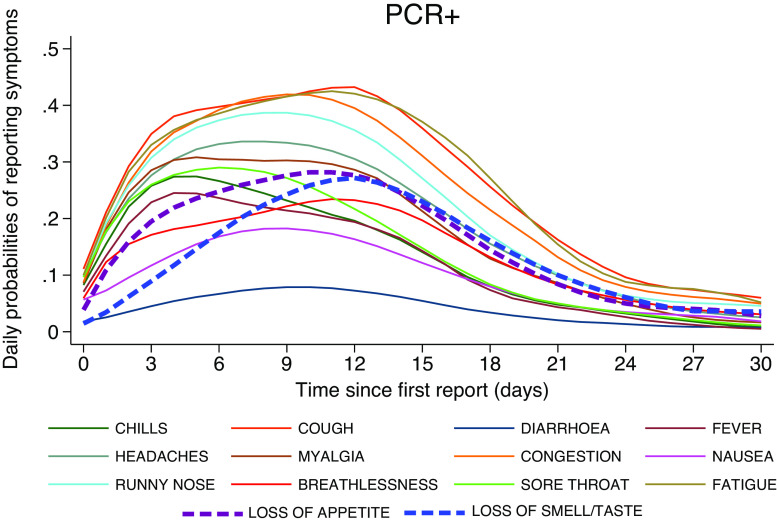
Daily probabilities of reporting specific symptoms starting with the first report conditioned on PCR+ participants and their corresponding illness episode, that is, ignoring the symptomatic episodes associated with these participants which were PCR-. Non-parametric methodology was used to capture the shape of the individual longitudinal daily reports.

**Figure 6.**
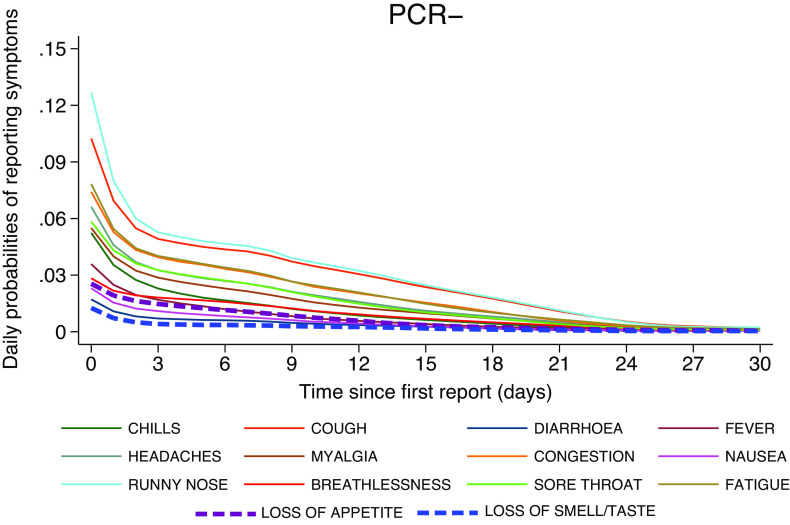
Daily probabilities of reporting specific symptoms starting with the first report using PCR- symptomatic episodes across all participants.

**Figure 7.**
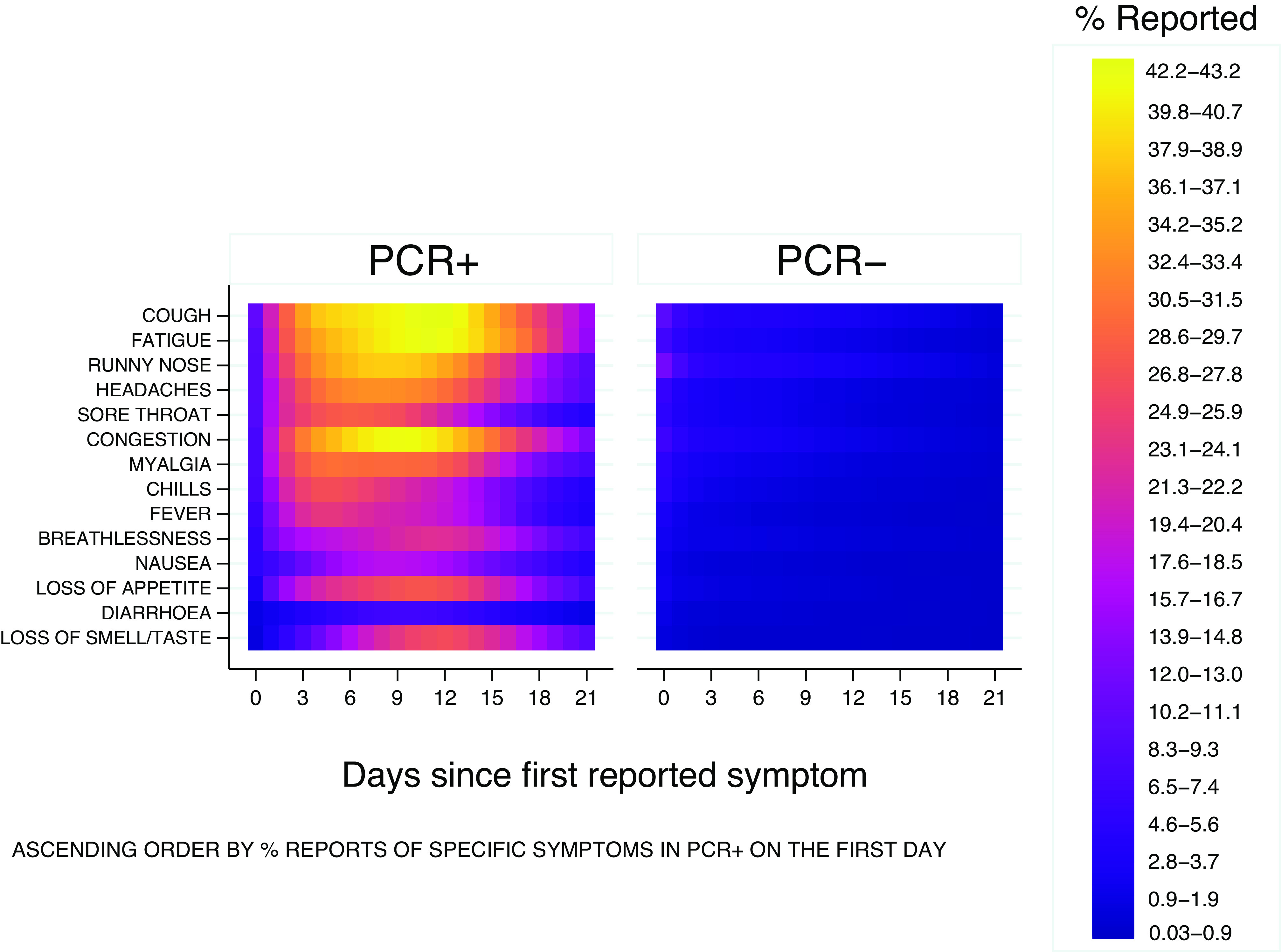
Probabilities of daily occurrences of various symptoms have similar magnitude in both PCR+ and PCR− groups on the first reporting day whilst they peak up later during illness evolution in PCR+ patients and decline in those PCR−, also reflected in previous figures 5 and 6.

Figures 5 and 6 present the daily probabilities of specific symptoms (starting with the first report of any symptom), stratified by PCR result. Whilst these probabilities fall swiftly in PCR− participants (Figure 6), they start more slowly and peak later in those with COVID-19 (Figure 5). Fever peaked on the 4th day (24%), followed by chills (27%), whilst myalgia (31%) and loss of appetite (28%) peaked on the 5th day. Anosmia/ageusia (27%) and cough (43%) peaked on the 12th day. These findings are also reflected in Figure 7; symptoms in PCR− participants fall rapidly shown by the dark purple, whereas they are later to peak and slower to fade in PCR+ participants, shown by the changing colour scale.

### The optimal diagnostic model for testing PCR+ based on symptoms and controlled for population characteristics

**Figure 8.**
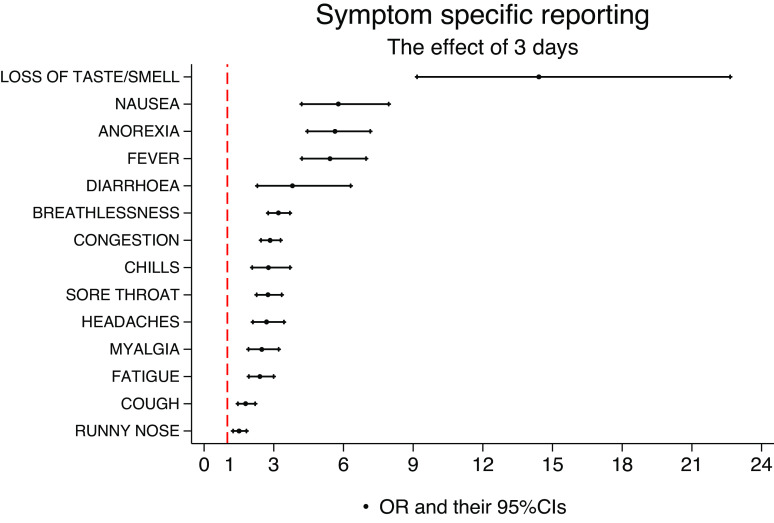
Effect (OR) of reporting a specific symptom for 3 days during an episode, irrespective of other symptoms reported during that episode.

Figure 8 presents the effects (ORs) of reporting a specific symptom for 3 days within an episode, on the probability of testing PCR+. The rationale for considering the 3-day symptom effect as a meaningful magnitude for the length of reports was inspired by Figure 4. In this figure, all specific symptoms seem to have a mean of less than 3 days in PCR− participants. Anosmia/ageusia (OR = 14.4 (95%CI 9.2, 22.6)), nausea (OR = 5.8 (95%CI 4.2, 7.9)), loss of appetite (OR = 5.6 (95%CI 4.5, 7.2)), and fever (OR = 5.4 (95%CI 4.2, 6.97)) have the strongest effects in terms of magnitude and statistical significance.

The most parsimonious model, that is, the model with the least number of predictors, yet explaining the most variability in the data, is shown in Table 5. The model retains anosmia/ageusia (OR = 5.2 (95%CI 3.4, 7.9)), loss of appetite (OR = 2.3 (95%CI 1.6, 3.3)), fever (OR = 1.9 (95%CI 1.4, 2.6)), congestion (OR = 1.9 (95%CI 1.5, 2.4)), and cough (OR = 1.3 (95%CI 1.1, 1.6)) as key symptoms associated with a PCR+ episode, whilst runny nose (OR = 0.7 (95%CI 0.5, 0.9)) and chills (OR = 0.6 (95%CI 0.4, 0.8)) are associated with testing PCR−. This model has a discrimination power of approximately 0.86 in terms of AUC but does not account for population weights.

**Table 5.** Optimal model for PCR+ based on symptoms and population characteristics on a two-level weighted logistic regression analysis

Variable	OR	*P*-value	95%CI low	95%CI high
Loss of taste and smell	5.181	0.000	3.400	7.894
Loss of appetite	2.323	0.000	1.643	3.283
Fever	1.880	0.000	1.385	2.552
Congestion	1.875	0.000	1.464	2.402
Cough	1.338	0.004	1.098	1.631
Runny nose	0.662	0.004	0.500	0.877
Chills	0.578	0.000	0.443	0.753
Age	0.988	0.024	0.977	0.998
BAME vs. white	2.434	0.001	1.406	4.214

The adjusted effects of three days of specific reports are shown.

Supplementary Material presents combinations of symptoms predicting the probabilities of COVID-19 using the optimal model. For example, a white participant of 50 years of age would have over 90% probability of testing PCR+ if s/he reported 3 days of loss of taste and smell, 3 days of loss of appetite, 3 days of fever, and 3 days of cough with 1 day of congestion, runny nose, and chills.

### The discriminatory power of specific symptoms

**Figure 9.**
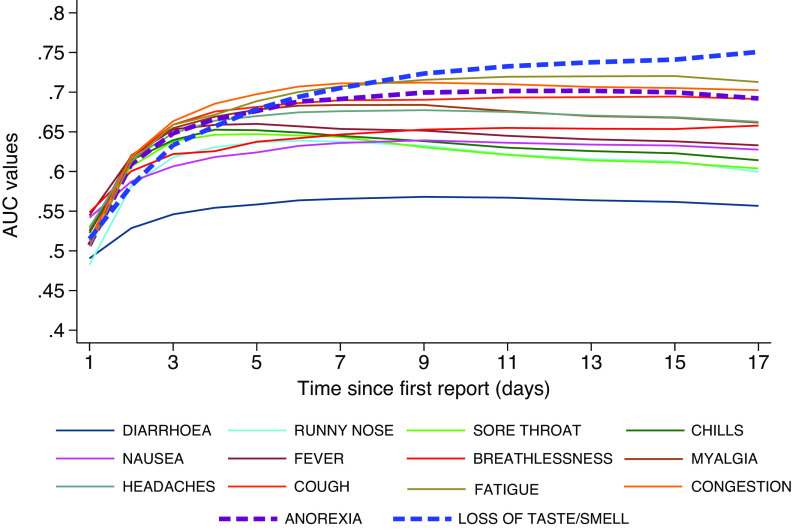
Discrimination power of individual symptoms based on the temporally ordered reports restricted to the first 1, 2, 3 to longer than 15 days after the symptomatic illness episode starts.

Figure 9 shows how the discriminatory power of individual symptoms evolves if only the first number of days after onset is considered – that is only day 1, only days 1–2, only days 1–3, and so on. Symptoms that peak later such as anosmia/ageusia gain discrimination power as the number of days of reporting increases. For other less specific symptoms, the individual discrimination power remains constant or even declines, for example, sore throat peaks very early and then tapers off.

**Figure 10.**
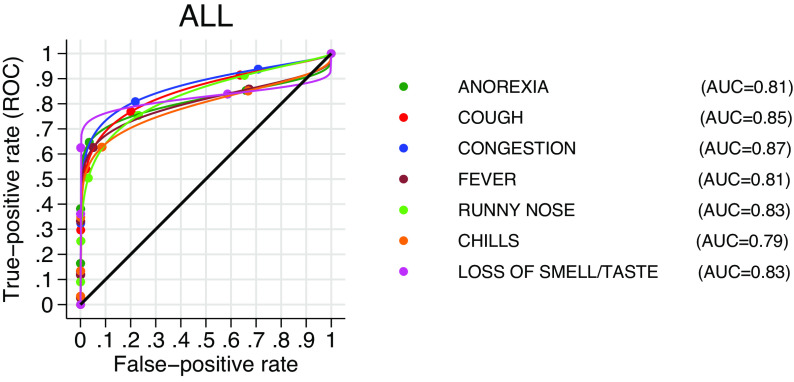
Estimated discrimination power of each classifier. The plot and the AUC estimate follow a maximum likelihood ROC-weighted regression analysis uncontrolled for age and ethnicity.

The area under the curve in Figure 10 shows the discrimination power of each symptom in the model using the maximum likelihood ROC 2-stage regression analysis (uncontrolled for age and ethnicity and population-weighted). The higher the AUC, the better the symptom discriminates between PCR+ and PCR−, the steep incline of the curve followed by the flattening line suggests that discrimination is little affected as the number of false positives increases.

**Figure 11.**
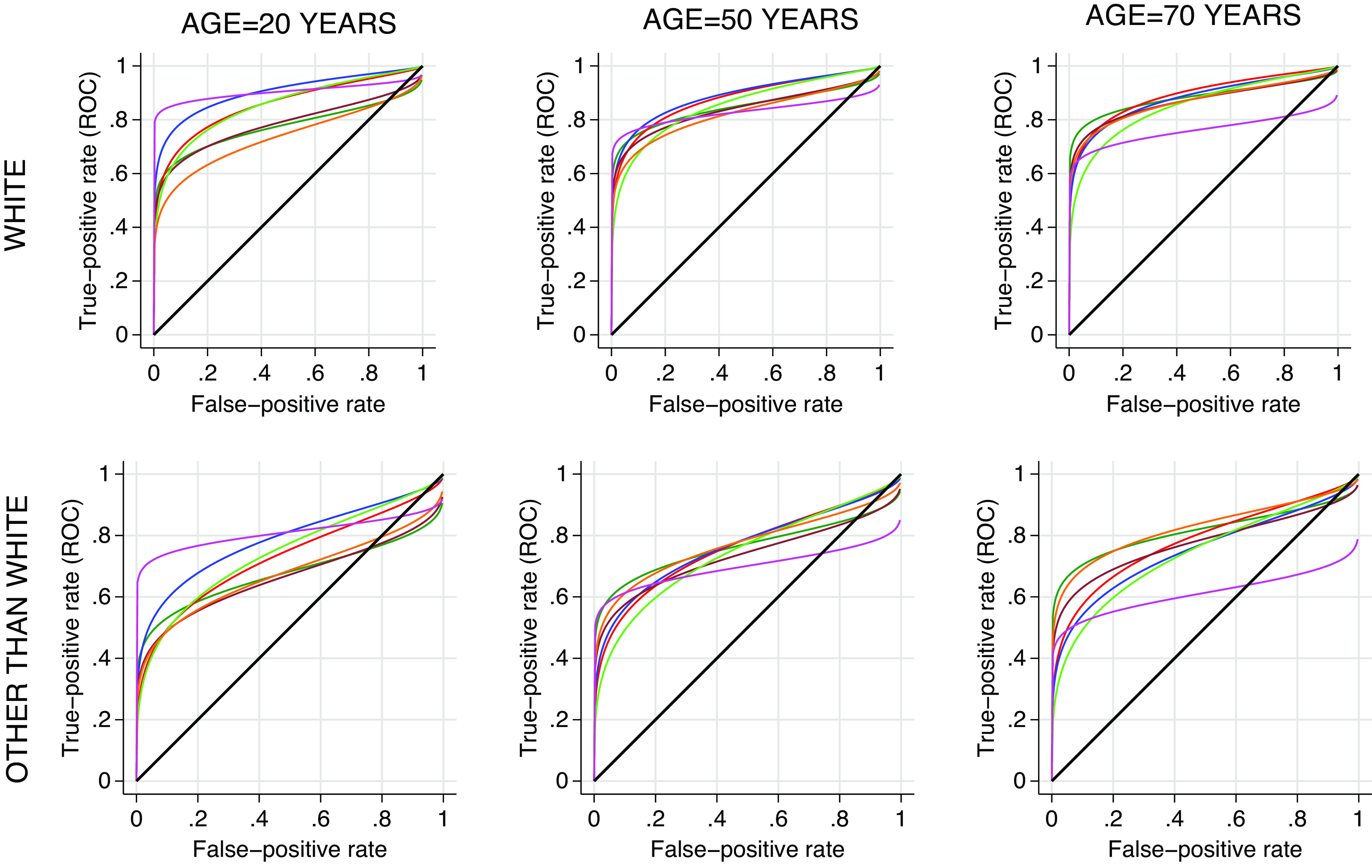
Effect of age and ethnicity on the ROC curve and subsequently on discrimination power associated with each classifier in the model. The colours indicating specific symptom are similar to those displayed in Figure 10.

When controlled for age and ethnicity, the two-stage ROC model does not quantify their effects on the ROC curve of specific symptoms in a directly interpretable manner, but qualitative conclusions are displayed in Table 6 and visualized in Figure 11. Age and ethnicity affect the ROC curve for each symptom, notably, the discriminatory power of anosmia/ageusia decreased with increasing age and is smaller ethnic minorities, compared to white ethnicity.

**Table 6.** Effect of age and ethnicity on the ROC curve and subsequently on discrimination power associated with each classifier in the model.

Symptoms		Coefficient	*P*-value	95%CI low	95%CI high
Loss of taste/smell	BAME vs. white	−0.436	**0.041**	−0.853	−0.019
	Age	−0.012	**0.011**	−0.021	−0.003
Anorexia	BAME vs. white	−0.312	0.116	−0.701	0.077
	Age	0.009	0.053	0.000	0.018
Fever	BAME vs. white	−0.390	**0.040**	−0.761	−0.018
	Age	0.007	0.109	−0.002	0.016
Congestion	BAME vs. white	−0.556	**0.016**	−1.007	−0.105
	Age	−0.003	0.583	−0.012	0.007
Cough	BAME vs. white	−0.521	**0.028**	−0.986	−0.055
	Age	0.004	0.408	−0.005	0.014
Runny nose	BAME vs. white	−0.467	**0.034**	−0.897	−0.036
	Age	0.000	0.998	−0.009	0.009
Chills	BAME vs. white	−0.191	0.316	−0.564	0.182
	Age	0.010	**0.023**	0.001	0.019

The coefficients are only qualitatively interpreted.

## Discussion

The main objectives of this study were to develop a symptom-based diagnostic model for a PCR− proven SARS-CoV-2 infection, and investigate the dynamics of the symptoms and their discrimination power for a potential COVID-19 diagnostic model. Our prospective, longitudinal, real-time collection, together with analytical techniques (post-stratification weights [20–22]), which produce generalizable results for the UK adult community population, provides a better understanding of the dynamics of COVID-19 symptomology. The rather poor engagement of people other than white in COVID-19 clinical trials has been documented [25] but our method overcame this difficulty.

We found a 4-month prevalence of COVID-19 of 2.1%, in line with the estimated population prevalence at that time [26]. Of the individual symptoms, anosmia and/or ageusia were the least reported symptoms overall (3.6%); however, participants reporting them for 3 days were more likely to test positive for COVID-19 (OR = 14.4 (95%CI 9.2, 22.6)). Figure 3 presents the probabilities of testing positive conditioned on symptom reports. Also, of those testing positive for SARS-CoV-2, over half (53.3%) reported the presence of anosmia or ageusia (Figure 2). Other symptoms such as loss of appetite, a new fever, congestion, and cough were strongly associated with a positive result. Fever, cough, and anosmia/ageusia have been identified as the strongest candidates for predicting COVID-19 in studies such as a REACT-1 and also in a meta-analysis of 9 studies examining symptoms of COVID-19 and long COVID syndrome [13, 17]. The odds of having COVID-19 have been reported as positively associated with 3 days reported with shortness of breath (OR = 3.1, (95%CI(2.9, 3.3)), although our results do not support it as a ‘leading’ symptom [13]. On its own runny nose was the most reported symptom (16.9%) in our study, and frequently reported in those with confirmed COVID-19 (72.6%). The participants reporting it were the least likely (8%) to test positive for COVID-19 (Figure 3), when accounting for the entire episode, and the symptom turned out to have high discriminatory power (AUC = 0.83, Figure 9) in ruling out the disease, consistent with other findings [11, 17].

Unlike many other studies [6–8, 10, 16], this research examined the number of days that specific symptoms are reported within an infection episode. We found that PCR+ participants reported a significantly longer duration of specific symptoms per episode, compared with those that were PCR−; cough had the longest duration followed by tiredness whilst runny nose had the longest duration among PCR− participants. We also found that cough, anosmia/ageusia, and loss of appetite peaked later in SARS-CoV-2 infection, typically around day 12 (Figure 5). Research in Czechoslovakia demonstrated anosmia and ageusia had a later onset than other symptoms, beginning a median of two or more days after the onset of symptoms, and lasting longer than fever or loss of appetite [27]. These findings are consistent with Wojtusiak et al. who found that headaches, chills, and cough were more relevant if they occurred at onset, whilst loss of taste and smell and loss of appetite had a higher relevance if they occurred later in the infection [12].

Previous research has suggested that individual symptoms are not predictive of COVID-19 on their own. Our analysis has suggested that individual symptoms would not have had sufficient predictive power for COVID-19 early in their occurrence but that this would increase with the number of days in which they manifest (Figure 9). Hence, our final predictive model is based on specific symptomatic episodes, that is, their entire number of symptomatic days within an episode and adjusted for age and ethnicity. The model retained episodes of anosmia/ageusia, loss of appetite, fever, congestion, and cough as all positively associated with testing PCR+, together with runny nose, chills, and age as all negatively associated with testing PCR+ (Table 5) consistent with other findings [28]. The concept of 3 days as a meaningful magnitude for the length of reports was inspired by Figure 4, in which all symptoms had a mean of less than 3 days in PCR− participants. In light of this, this information may be particularly useful at the time of clinical triage, namely the number of days symptoms have been experienced by subjects presenting for hospital care. The model, based on two-level logistic regression, has a discriminating power of ~86%.

Our ROC analysis showed that the discrimination power of anosmia/ageusia increased from irrelevance during the first few days to exceeding all others after day 9 (Figure 9). Our report also showed that the discriminatory power of anosmia/ageusia decreases with age, which may reflect a biological phenomenon associated with ageing [29]. Cough alone remained relatively constant in its discrimination power, however, PCR− participants also reported prolonged cough. Our data do not support diarrhoea as a candidate symptom of COVID-19.

Two-stage ROC analysis suggests that the prediction power may be less discriminatory in older participants and in those from ethnic minorities, this was true for all symptoms. Comparatively, the Canas et al. model showed better discrimination in participants of normal weight compared to those who were underweight and/or overweight, and in non-healthcare workers and, consistent with our results, found that younger people were more likely to test PCR+, possibly due to increased social mixing [15]. Our diagnostic model is similar to this model as it identified persistent cough and loss of smell, alongside abdominal pain and myalgia as early features of COVID-19 [15]. However, the Canas model had a younger population than our study (mean age 46.7 years vs. 53.1 years) and COVID-19 was self-reported, thereby the results are difficult to compare [15]. Moreover, the study reported ‘blisters on the feet’ and ‘eye soreness’ as relevant features of COVID-19, the significance of which the paper questions itself [15].

Our estimated prevalences of specific symptoms among both positive and negative groups are higher than those presented in the meta-analysis by Bowyer et al. [13]. Although the study participants stem from nine longitudinal cohorts, the data collection is essentially retrospective and cross-sectional. The authors stated a great deal of heterogeneity. Notably, the data have been collected during the summer whilst ours were collected during the winter, including Christmas, when transmission intensified, hence we postulate that variation could be attributable to the season. Our prevalence of specific symptoms among PCR+ and PCR− are closest to those from Generation Scotland cohort (access via Bowyer et al. or from the University of Edinburgh) [13, 30] consistent with our explanation above, given somewhat cooler temperatures in Scotland during the summer. We have retrieved some partial information and appended a relevant comparative table in the Supplementary material.

Though multiple centres participated in the clinical trial, the three-level regression techniques did not reveal important differences in the estimates or their standard errors. Variability between the centres was not expected to be significant as the same trial protocol and procedures were used. We have disregarded the effect of the intervention (placebo or vaccine), as preliminary analysis did not show a significant impact on results (data not shown).

## Limitations

Despite the data being gathered prospectively and in real-time, we observed gaps in daily records, for example, a participant may report fever for 3 consecutive days, then none on the fourth day and then again on the fifth and sixth days. The statistical analysis considered the number of reports (i.e. the number of days with specific symptoms) rather than the whole length of time they were experienced. This may have led to underestimating their effect; however, we are confident that recall bias has been minimalized to a greater extent than if the data had been collected from a retrospectively collected self-report. Asymptomatic infections are likely to be underrepresented in this analysis. As this research set out to explore symptoms of COVID-19, we do not believe this to be a major limitation to our analysis, but it does mean we cannot calculate the true prevalence of COVID-19 infections in the study population. Unfortunately, we also did not benefit from information such as recent contacts or travel/work patterns, which could have been useful in building a reliable diagnostic model as suggested by the Cochrane Review article [10]. At the time of data collection, the circulating strain of SARS-CoV-2 was the alpha variant [31], however, omicron has a higher tropism for nasoepithelial cells than pulmonary cells [32] and anosmia has been reported less frequently with the omicron variant [33]. Therefore, care should be taken if applying the model outside our study population.

## Conclusion

This research adds to the body of literature on COVID-19 symptoms as an in-depth exploration of symptoms reported by those unaware of their diagnosis at the time of reporting, thereby minimizing reporting bias. We found younger participants, and those from ethnic minorities were more likely to test positive for COVID-19 and, consistent with previous research, anosmia and/or ageusia most strongly predict a positive PCR result; however, we have also shown that these symptoms peak late in infection. This calls into question their consideration as early markers of the disease. Similar to other research we found that a cluster of fever, congestion, and cough are all positively associated with COVID-19, with PCR+ participants reporting more days of symptoms, for example, cough, than those who were PCR−. We also found that diarrhoea, runny nose, and chills are not indicative of COVID-19. Overall, our model has a discriminating power of 86% to predict COVID-19; although, as anosmia and ageusia often develop later in the infection, our proposed model is unlikely to identify early infections, particularly, in the elderly or those from ethnic minorities.

## Supporting information

Bird et al. supplementary material Bird et al. supplementary material

## Data Availability

The data are available upon request and subject to Novavax’s permission. Please contact Professor Paul T. Heath, pheath@sgul.ac.uk.

## Supplementary material

For supplementary material accompanying this paper visit http://doi.org/10.1017/S0950268824000037. click here to view supplementary material
