# Supplementary material for: The predictive role of symptoms in COVID-19 diagnostic models: A longitudinal insight
Source: Epidemiol Infect. 2024 Jan 22;152:e37. doi: 10.1017/S0950268824000037 (PMC10945957; doi:10.1017/S0950268824000037)
Supplement: Bird et al. supplementary material [file S0950268824000037sup001.docx]

# Supplementary material

## Data collection

We have used the UK phase 3 double blind clinical trial of the efficacy and safety of the NVX-CoV2373 vaccine (Novavax), collected from the start of recruitment (28^th^ November 2020) until the end of February 2021. Data on demographics (age, gender, ethnicity, BMI, obesity, and comorbidities), dates of reported symptoms (based on an *a priori* established candidate list of symptoms and dates of PCR testing were pooled from the clinical trial database and linked according to participant’s anonymized identification code and dates of events.

## Data structure and master file construction

The collected data had multiple layers of hierarchy, and this has implications on the sources of variability which needs to be accounted for in the analyses for correct p-values and standard errors.

Data on demographics (age, gender, ethnicity, BMI, obesity, and comorbidities), dates of reported symptoms and dates of PCR testing were pooled from the clinical trial database and linked according to participant’s anonymized identification code and dates of events, i. e. symptoms reports or PCR tests.

A master spreadsheet was then created with multiple event-episodes per participant which included symptoms from the designated list and/or the results of a PCR test. Each event-episode was assigned a *binary indicator* *for the presence/absence of a specific symptom*, a number indicating the *number of specific symptoms* and a *binary indicator for the PCR status* during that potential episode. As such, participants could have multiple symptomatic episodes which could be PCR positive or negative. Any symptoms occurring within seven days of the vaccinations were excluded, unless the participant was PCR positive.

We have built a hierarchical data set with multiple episodes associated with each participant, allowing for the possibility of more than one event episode and its associated PCR confirmed test result.

## Descriptive statistics

Descriptive statistics and graphics on cross sectional variables (age, gender, ethnicity, BMI, obesity as those with BMI>30, and comorbidities) were used to understand their distribution overall and across a simple binary outcome indicating a PCR positive test or not. Continuous variables are summarized by their means, medians, standard deviations, inter-quartiles as well as the number of observations these summaries rely on. Categorical/binary are summarized as proportions and missing data have been assessed. Summary statistics are presented overall and by the PCR status.

## Population weights

To extrapolate the results to the UK population we plotted and empirically compared the distribution of age, gender and ethnicity distributions in the sample data to that of the UK population^16–18^. We then used post-stratification techniques for incorporating population demographic distributions (1). This technique allowed us to migrate from a randomised clinical trial setting to an observational epidemiological study representative for the UK population. Weights were derived and assigned to each participant such that the subsequent estimation procedures inflated the effect of under-represented groups (e.g. young BAME) and depressed the effect of overrepresented groups in the sample (e.g. old White).

The sample data frequencies have been calculated by stratifying the participants by 5-year age groups, gender and ethnicity, resulting in 13×2×2=52 strata corresponding to 13 age groups, 2 to gender and 2 to ethnicity (White vs. BAME). Similar stratified frequencies have been derived from ONS data and then weights corresponding to each stratum have been calculated as $\frac{{stratum prop}_{population}}{{stratum prop}_{sample}}$ . Each participant has been assigned a weight according to the stratum to which s/he belongs. The sum of all individual weights equals the number of participants.

## Analytical strategy

The first step was to develop a general, simplified picture of the data by cross-tabulating participants with at least one symptomatic episode and those with no symptomatic episodes against participants who tested positive at least once or never.

Participants were grouped by their symptoms, one group who “reported a specific symptom” and another who “never reported that symptom”, with further stratification by PCR status: either positive or negative. We have graphically depicted the proportions of PCR+ among participants reporting specific symptoms, irrespective of their number and of whether they reported other symptoms. We have also illustrated the proportions of participants with at least one specific symptom, overall and conditioned on testing ever PCR+ or always PCR-. Using this binary classification for specific episodes we derived the predicted probabilities of a PCR+ test conditioned on each specific symptom using a population-weighted binary regression with robust standard errors to allow for cluster-defined multiple episodes associated with a participant. A two-level population-weighted logistic regression and more advanced lasso techniques produced similar results despite the low proportion of cases; hence we have preferred the former for simplicity and relatability with a wider audience.

The next analytical strategy investigated the specific symptom episodes in associations with the PCR+, not only for their occurrence as before but also in terms of number of reports (recorded once per day) associated with each type of symptom within an event-episode. At this stage, we have also investigated the number of days of specific symptoms reported within an episode as an outcome to understand their variability by population characteristics. That revealed a series of important potential confounders candidates for the associations between the PCR binary outcome and the number of specific symptoms, namely to understand which aspects of the population are related to both these variables and influence for the Receiver operating characteristic (ROC) analyses. We have used a two-level zero-inflated Poisson regression for count data that has an excess of zero counts given the large number of participants with no symptoms.

Finally, prior to building an optimal model, we assessed the effect of reporting each specific symptom for 3 days on the probability of testing PCR+ vs PCR-, measured as the odds ratios and their 95% CIs. We have derived an optimal model, namely a model with the least number of variables which significantly explained the variability in the outcome, based on two-level logistic multivariable regression using forward-backward elimination and p-values less than 0.05 as criteria for keeping explanatory variables in the model. These included the number of specific symptoms and population characteristics such as age and ethnicity as potential confounders. We have also evaluated the discriminatory power of this model using its associated ROC curve and its area under the curve (AUC) as a metric for this discrimination.

In addition, we also performed a two-stage process ROC, i.e. ROC regression (2). The advantage of this analysis over the logistic regression is that the ROC curve can be controlled and illustrated across groups in the population upon which its discrimination accuracy may vary according to age and ethnicity^15^. Each symptom could be evaluated separately for its discrimination power, uncontrolled first and then controlled for age and ethnicity. The result of the latter version is an estimate of the ROC curve for each specific symptom as as a function of age and ethnicity -known as covariate specific ROC curve. The techniques accommodated for multiple episodes associated with an individual and for adjustments using population weights.

Finally, non-parametric techniques such as local polynomial smoothing have been used to fit curves on the daily probabilities of the reports in the PCR+ and PCR- participants as it was not possible to capture all the inflection points and curvatures for each or all symptoms series in a systematic parametric manner. Nevetheless, the tool accurately highlights different dynamics of recorded symptoms by the days of reporting, starting with the first day in each group defined by the PCR status.

All the analyses and graphical plots have been perfomed in Stata (StataCorp. 2021. *Stata Statistical Software: Release 17*. College Station, TX: StataCorp LLC) and (R Statistical Software (v4.1.2; R Core Team 2021)).

1. R. J. A. Little, Post-Stratification: A Modeler's Perspective, Journal of the American Statistical Association

Vol. 88, No. 423 (Sep., 1993), pp. 1001-1012

1. Alonzo, T. A. & Pepe, M. S. Distribution-free ROC analysis using binary regression techniques. Biostatistics, **2020**; 3: 421–432

| **PCR positive** | | | | | | | | | | | | | | | | | | | | | | | | | | | | | | | | | | | | | | | | | |
| --- | --- | --- | --- | --- | --- | --- | --- | --- | --- | --- | --- | --- | --- | --- | --- | --- | --- | --- | --- | --- | --- | --- | --- | --- | --- | --- | --- | --- | --- | --- | --- | --- | --- | --- | --- | --- | --- | --- | --- | --- | --- |
| 1 |  | 2 |  | 3 |  | 4 |  | 5 |  | 6 |  | 7 |  | 8 |  | 9 |  | 10 |  | 11 |  | 12 |  | 13 |  | 14 |  | 15 |  | 16 |  | 17 |  | 18 |  | 19 |  | 20 |  | 21 |  |
| .015 | SM/TAS | .025 | DIARRH | .035 | DIARRH | .045 | DIARRH | .054 | DIARRH | .061 | DIARRH | .067 | DIARRH | .073 | DIARRH | .077 | DIARRH | .079 | DIARRH | .079 | DIARRH | .077 | DIARRH | .073 | DIARRH | .068 | DIARRH | .062 | DIARRH | .054 | DIARRH | .047 | DIARRH | .040 | DIARRH | .034 | DIARRH | .029 | DIARRH | .024 | DIARRH |
| .018 | DIARRH | .034 | SM/TAS | .062 | SM/TAS | .089 | SM/TAS | .117 | SM/TAS | .146 | SM/TAS | .168 | NAUSEA | .177 | NAUSEA | .182 | NAUSEA | .182 | NAUSEA | .179 | NAUSEA | .173 | NAUSEA | .163 | NAUSEA | .151 | NAUSEA | .136 | NAUSEA | .122 | NAUSEA | .107 | NAUSEA | .092 | FEVER | .074 | FEVER | .059 | FEVER | .051 | FEVER |
| .039 | APETTITE | .073 | NAUSEA | .096 | NAUSEA | .117 | NAUSEA | .137 | NAUSEA | .155 | NAUSEA | .175 | SM/TAS | .202 | SM/TAS | .212 | BREATH | .214 | FEVER | .209 | FEVER | .201 | FEVER | .194 | FEVER | .180 | CHILLS | .163 | CHILLS | .141 | CHILLS | .119 | CHILLS | .093 | NAUSEA | .080 | NAUSEA | .066 | CHILLS | .055 | CHILLS |
| .057 | NAUSEA | .109 | APETTITE | .155 | BREATH | .171 | BREATH | .181 | BREATH | .188 | BREATH | .195 | BREATH | .203 | BREATH | .220 | FEVER | .222 | BREATH | .220 | CHILLS | .207 | CHILLS | .196 | CHILLS | .181 | FEVER | .166 | FEVER | .143 | FEVER | .119 | FEVER | .097 | CHILLS | .080 | CHILLS | .068 | NAUSEA | .057 | NAUSEA |
| .059 | BREATH | .123 | BREATH | .159 | APETTITE | .195 | APETTITE | .219 | APETTITE | .236 | APETTITE | .236 | FEVER | .227 | FEVER | .225 | SM/TAS | .232 | CHILLS | .230 | BREATH | .234 | BREATH | .217 | THROAT | .195 | THROAT | .171 | THROAT | .148 | THROAT | .124 | THROAT | .103 | THROAT | .084 | THROAT | .069 | THROAT | .057 | THROAT |
| .071 | FEVER | .134 | FEVER | .191 | FEVER | .229 | FEVER | .245 | FEVER | .244 | FEVER | .248 | APETTITE | .256 | CHILLS | .245 | CHILLS | .243 | SM/TAS | .257 | THROAT | .238 | THROAT | .232 | BREATH | .226 | BREATH | .214 | BREATH | .197 | BREATH | .175 | BREATH | .153 | BREATH | .129 | MYALG | .113 | MYALG | .097 | BREATH |
| .083 | CHILLS | .155 | CHILLS | .221 | CHILLS | .258 | CHILLS | .274 | CHILLS | .274 | CHILLS | .266 | CHILLS | .259 | APETTITE | .268 | APETTITE | .272 | THROAT | .259 | SM/TAS | .268 | SM/TAS | .272 | SM/TAS | .264 | SM/TAS | .243 | MYALG | .213 | MYALG | .183 | MYALG | .154 | MYALG | .132 | BREATH | .113 | BREATH | .099 | MYALG |
| .086 | MYALG | .176 | HDACHE | .230 | THROAT | .260 | THROAT | .277 | THROAT | .286 | THROAT | .290 | THROAT | .288 | THROAT | .282 | THROAT | .276 | APETTITE | .282 | APETTITE | .282 | APETTITE | .276 | APETTITE | .266 | APETTITE | .246 | APETTITE | .222 | APETTITE | .198 | APETTITE | .171 | APETTITE | .144 | APETTITE | .122 | APETTITE | .102 | APETTITE |
| .088 | NASAL | .178 | THROAT | .235 | HDACHE | .276 | HDACHE | .303 | MYALG | .308 | MYALG | .305 | MYALG | .304 | MYALG | .302 | MYALG | .303 | MYALG | .301 | MYALG | .296 | MYALG | .286 | MYALG | .271 | MYALG | .249 | SM/TAS | .230 | SM/TAS | .209 | SM/TAS | .181 | HDACHE | .155 | HDACHE | .135 | HDACHE | .116 | HDACHE |
| .097 | THROAT | .181 | MYALG | .246 | MYALG | .285 | MYALG | .305 | HDACHE | .322 | HDACHE | .332 | HDACHE | .336 | HDACHE | .336 | HDACHE | .334 | HDACHE | .329 | HDACHE | .319 | HDACHE | .305 | HDACHE | .287 | HDACHE | .263 | HDACHE | .237 | HDACHE | .210 | HDACHE | .184 | SM/TAS | .161 | SM/TAS | .139 | SM/TAS | .118 | SM/TAS |
| .098 | HDACHE | .188 | NASAL | .259 | RUNNY | .307 | RUNNY | .340 | RUNNY | .361 | RUNNY | .374 | RUNNY | .383 | RUNNY | .387 | RUNNY | .387 | RUNNY | .382 | RUNNY | .372 | RUNNY | .356 | RUNNY | .334 | RUNNY | .304 | RUNNY | .271 | RUNNY | .238 | RUNNY | .203 | RUNNY | .170 | RUNNY | .145 | RUNNY | .122 | RUNNY |
| .098 | RUNNY | .192 | RUNNY | .264 | NASAL | .319 | NASAL | .352 | NASAL | .373 | NASAL | .386 | TIRED | .398 | TIRED | .407 | TIRED | .416 | COUGH | .418 | NASAL | .410 | NASAL | .395 | NASAL | .373 | NASAL | .344 | NASAL | .311 | NASAL | .277 | NASAL | .245 | NASAL | .215 | NASAL | .188 | NASAL | .160 | NASAL |
| .100 | TIRED | .201 | TIRED | .283 | TIRED | .330 | TIRED | .357 | TIRED | .374 | TIRED | .392 | NASAL | .404 | COUGH | .410 | COUGH | .416 | TIRED | .422 | TIRED | .425 | TIRED | .421 | TIRED | .411 | TIRED | .392 | COUGH | .360 | COUGH | .325 | COUGH | .291 | COUGH | .256 | COUGH | .223 | COUGH | .190 | TIRED |
| .111 | COUGH | .211 | COUGH | .292 | COUGH | .350 | COUGH | .381 | COUGH | .391 | COUGH | .398 | COUGH | .407 | NASAL | .415 | NASAL | .419 | NASAL | .425 | COUGH | .431 | COUGH | .432 | COUGH | .416 | COUGH | .394 | TIRED | .371 | TIRED | .343 | TIRED | .311 | TIRED | .271 | TIRED | .230 | TIRED | .192 | COUGH |
| **PCR negative** | | | | | | | | | | | | | | | | | | | | | | | | | | | | | | | | | | | | | | | | | |
| .013 | SM/TAS | .007 | SM/TAS | .005 | SM/TAS | .004 | SM/TAS | .004 | SM/TAS | .004 | SM/TAS | .004 | SM/TAS | .003 | SM/TAS | .003 | SM/TAS | .003 | SM/TAS | .003 | SM/TAS | .003 | SM/TAS | .003 | SM/TAS | .002 | SM/TAS | .002 | SM/TAS | .002 | SM/TAS | .001 | SM/TAS | .001 | SM/TAS | .001 | SM/TAS | .001 | SM/TAS | .001 | SM/TAS |
| .017 | DIARRH | .011 | DIARRH | .008 | DIARRH | .007 | DIARRH | .007 | DIARRH | .006 | DIARRH | .006 | DIARRH | .006 | DIARRH | .005 | DIARRH | .005 | DIARRH | .004 | DIARRH | .004 | DIARRH | .004 | DIARRH | .003 | DIARRH | .003 | DIARRH | .003 | DIARRH | .002 | DIARRH | .002 | DIARRH | .002 | DIARRH | .001 | DIARRH | .001 | DIARRH |
| .023 | NAUSEA | .015 | NAUSEA | .012 | NAUSEA | .011 | NAUSEA | .010 | NAUSEA | .009 | NAUSEA | .008 | NAUSEA | .008 | NAUSEA | .007 | NAUSEA | .006 | NAUSEA | .005 | NAUSEA | .005 | NAUSEA | .004 | NAUSEA | .004 | NAUSEA | .003 | NAUSEA | .003 | NAUSEA | .002 | NAUSEA | .002 | NAUSEA | .002 | NAUSEA | .002 | NAUSEA | .001 | NAUSEA |
| .025 | APETTITE | .019 | APETTITE | .016 | APETTITE | .015 | APETTITE | .013 | APETTITE | .012 | APETTITE | .011 | APETTITE | .011 | APETTITE | .009 | FEVER | .008 | FEVER | .007 | FEVER | .006 | FEVER | .006 | FEVER | .005 | APETTITE | .004 | APETTITE | .003 | APETTITE | .003 | APETTITE | .003 | APETTITE | .002 | APETTITE | .002 | APETTITE | .002 | APETTITE |
| .028 | BREATH | .022 | BREATH | .019 | BREATH | .017 | FEVER | .015 | FEVER | .014 | FEVER | .012 | FEVER | .011 | FEVER | .010 | APETTITE | .008 | APETTITE | .007 | APETTITE | .007 | APETTITE | .006 | APETTITE | .005 | FEVER | .005 | FEVER | .004 | FEVER | .003 | FEVER | .003 | FEVER | .003 | FEVER | .002 | FEVER | .002 | FEVER |
| .036 | FEVER | .025 | FEVER | .019 | FEVER | .018 | BREATH | .017 | BREATH | .017 | BREATH | .016 | BREATH | .015 | BREATH | .014 | BREATH | .012 | CHILLS | .011 | CHILLS | .010 | CHILLS | .009 | CHILLS | .008 | CHILLS | .007 | CHILLS | .006 | CHILLS | .006 | CHILLS | .005 | CHILLS | .004 | CHILLS | .004 | CHILLS | .003 | CHILLS |
| .052 | CHILLS | .035 | CHILLS | .027 | CHILLS | .023 | CHILLS | .020 | CHILLS | .018 | CHILLS | .017 | CHILLS | .015 | CHILLS | .014 | CHILLS | .012 | BREATH | .011 | BREATH | .010 | BREATH | .009 | BREATH | .008 | BREATH | .007 | BREATH | .007 | BREATH | .006 | BREATH | .006 | BREATH | .005 | BREATH | .004 | BREATH | .004 | BREATH |
| .055 | MYALG | .040 | MYALG | .032 | MYALG | .029 | MYALG | .026 | MYALG | .025 | MYALG | .023 | MYALG | .021 | MYALG | .020 | MYALG | .017 | MYALG | .016 | MYALG | .014 | MYALG | .013 | MYALG | .012 | MYALG | .011 | MYALG | .010 | MYALG | .009 | MYALG | .008 | MYALG | .007 | MYALG | .006 | MYALG | .005 | THROAT |
| .058 | THROAT | .043 | THROAT | .036 | THROAT | .033 | HDACHE | .030 | HDACHE | .028 | HDACHE | .027 | HDACHE | .025 | THROAT | .023 | THROAT | .021 | THROAT | .019 | THROAT | .017 | THROAT | .015 | THROAT | .013 | THROAT | .012 | THROAT | .010 | THROAT | .009 | THROAT | .008 | THROAT | .007 | THROAT | .006 | THROAT | .005 | MYALG |
| .066 | HDACHE | .046 | HDACHE | .037 | HDACHE | .033 | THROAT | .030 | THROAT | .029 | THROAT | .027 | THROAT | .026 | HDACHE | .024 | HDACHE | .021 | HDACHE | .019 | HDACHE | .018 | HDACHE | .016 | HDACHE | .014 | HDACHE | .013 | HDACHE | .011 | HDACHE | .010 | HDACHE | .009 | HDACHE | .008 | HDACHE | .007 | HDACHE | .006 | HDACHE |
| .074 | NASAL | .053 | NASAL | .043 | NASAL | .039 | NASAL | .037 | NASAL | .035 | NASAL | .033 | NASAL | .032 | NASAL | .029 | NASAL | .027 | NASAL | .024 | NASAL | .022 | NASAL | .020 | NASAL | .019 | NASAL | .017 | TIRED | .015 | TIRED | .013 | TIRED | .011 | TIRED | .010 | TIRED | .009 | TIRED | .007 | NASAL |
| .078 | TIRED | .055 | TIRED | .044 | TIRED | .040 | TIRED | .038 | TIRED | .036 | TIRED | .034 | TIRED | .032 | TIRED | .030 | TIRED | .027 | TIRED | .025 | TIRED | .023 | TIRED | .021 | TIRED | .019 | TIRED | .017 | NASAL | .015 | NASAL | .014 | NASAL | .012 | NASAL | .010 | NASAL | .009 | NASAL | .008 | TIRED |
| .102 | COUGH | .069 | COUGH | .055 | COUGH | .049 | COUGH | .047 | COUGH | .045 | COUGH | .044 | COUGH | .043 | COUGH | .040 | COUGH | .037 | COUGH | .035 | COUGH | .033 | COUGH | .031 | COUGH | .028 | COUGH | .026 | COUGH | .024 | COUGH | .022 | COUGH | .020 | COUGH | .018 | COUGH | .015 | COUGH | .013 | COUGH |
| .127 | RUNNY | .080 | RUNNY | .060 | RUNNY | .053 | RUNNY | .050 | RUNNY | .048 | RUNNY | .047 | RUNNY | .045 | RUNNY | .043 | RUNNY | .039 | RUNNY | .037 | RUNNY | .035 | RUNNY | .032 | RUNNY | .030 | RUNNY | .027 | RUNNY | .025 | RUNNY | .022 | RUNNY | .020 | RUNNY | .018 | RUNNY | .016 | RUNNY | .014 | RUNNY |

Supplementary Table 1: Daily probabilities reported by the participants in the descending order of their reporting in the PCR positive group. The table is related with Figures 5, 6 and 7.

|  | **OUR STUDY** | | | | **BOWYER et. al. meta-analysis study** | | |
| --- | --- | --- | --- | --- | --- | --- | --- |
|  | **PCR+** |  | **PCR-** | | **PCR+** | **PCR+** | **PCR+** |
| **SYMPTOMS** | **317** |  | **14822** | | **ALPACS** | **USoc** | **GS** |
|  | No | % | No | % | % | % | % |
| **RUNNY NOSE** | 230 | 72.6% | 2329 | 15.7% | 16% | 45.2% | **25.8%** |
| **COUGH** | 238 | 75.1% | 1967 | 13.3% | 3.8% | 37.3% | **50%** |
| **FATIGUE** | 236 | 74.4% | 1672 | 11.3% | 25% | 63.3% | **77%** |
| **CONGESTION** | 237 | 74.8% | 1641 | 11.1% |  |  |  |
| **HEADACHES** | 222 | 70.0% | 1496 | 10.1% | 14.4% | 52.9% | **63.5%** |
| **SORE THROAT** | 198 | 62.5% | 1397 | 9.4% | 9.4% | 38.7% | **31.5%** |
| **MYALGIA** | 206 | 65.0% | 1257 | 8.5% | 12.2% | 50.5% | **53.2%** |
| **CHILLS** | 189 | 59.6% | 1209 | 8.2% | 4.7% | 31.4% |  |
| **FEVER** | 196 | 61.8% | 932 | 6.3% | 4.5% | 31.3% | **45%** |
| **BREATHLESSNESS** | 162 | 51.1% | 783 | 5.3% | 6.3% | 32% | **39%** |
| **LOSS OF APPETITE** | 184 | 58.0% | 703 | 4.7% | 9.0 | 18.7% | **41.7%** |
| **NAUSEA** | 145 | 45.7% | 661 | 4.5% | 7.1 | 15.8% | **28.6%** |
| **DIARRHOEA** | 99 | 31.2% | 521 | 3.5% | 6.3% | 21.8% | **26.6%** |
| **ANOSMIA/AGEUSIA** | 169 | 53.3% | 372 | 2.5% | 6.8% | 31.3% | **49.6%** |

Supplementary Table 2: Comparative findings between our study with findings displayed in Table 4 and from 3 of the most comparable cohorts used in Bowyer et. al. meta-analysis study.

|  | **ALL** | | **PCR+** |  | **PCR-** | |
| --- | --- | --- | --- | --- | --- | --- |
| **SYMPTOMS** | **15139** | | **317** |  | **14822** | |
|  | Number | Proportion | Number | Proportion | Number | Proportion |
| **RUNNY NOSE** | 2559 | 16.9% | 230 | 72.6% | 2329 | 15.7% |
| **COUGH** | 2205 | 14.6% | 238 | 75.1% | 1967 | 13.3% |
| **FATIGUE** | 1908 | 12.6% | 236 | 74.4% | 1672 | 11.3% |
| **CONGESTION** | 1878 | 12.4% | 237 | 74.8% | 1641 | 11.1% |
| **HEADACHES** | 1718 | 11.3% | 222 | 70.0% | 1496 | 10.1% |
| **SORE THROAT** | 1595 | 10.5% | 198 | 62.5% | 1397 | 9.4% |
| **MYALGIA** | 1463 | 9.7% | 206 | 65.0% | 1257 | 8.5% |
| **CHILLS** | 1398 | 9.2% | 189 | 59.6% | 1209 | 8.2% |
| **FEVER** | 1128 | 7.5% | 196 | 61.8% | 932 | 6.3% |
| **BREATHLESSNESS** | 945 | 6.2% | 162 | 51.1% | 783 | 5.3% |
| **ANOREXIA** | 887 | 5.9% | 184 | 58.0% | 703 | 4.7% |
| **NAUSEA** | 806 | 5.3% | 145 | 45.7% | 661 | 4.5% |
| **DIARRHOEA** | 620 | 4.1% | 99 | 31.2% | 521 | 3.5% |
| **LOSS OF SMELL/TASTE** | 541 | 3.6% | 169 | 53.3% | 372 | 2.5% |

Table 4. Number (proportions) of participants with specific symptoms, overall and conditioned on the presence/absence of a PCR confirmed episode. The graphics representation of these proportions are presented in the Figure 2 of tha paper.

|  | **AGE** | | | | **GENDER** | | | | **ETHNICITY** | | | | **BMI** | | | | **COMORBIDITES** | | | |
| --- | --- | --- | --- | --- | --- | --- | --- | --- | --- | --- | --- | --- | --- | --- | --- | --- | --- | --- | --- | --- |
|  | RR | p-  value | 95%CI  Low-High | | RR | p-  value | 95%CI  Low-High | | RR | p-  value | 95%CI  Low-High | | RR | p-  value | 95%CI  Low-High | | RR | p-  value | 95%CI  Low-High | |
| **RUNNY NOSE** | 1.003 | 0.390 | 0.996 | 1.011 | 1.006 | 0.950 | 0.831 | 1.219 | 0.608 | 0.050 | 0.369 | 1.001 | 1.008 | 0.416 | 0.989 | 1.028 | 0.982 | 0.851 | 0.814 | 1.185 |
| **COUGH** | 1.005 | 0.134 | 0.999 | 1.011 | 1.070 | 0.449 | 0.898 | 1.275 | 0.681 | 0.063 | 0.455 | 1.020 | 1.016 | **0.022** | 1.002 | 1.030 | 1.151 | 0.092 | 0.977 | 1.355 |
| **FATIGUE** | 1.006 | 0.054 | 1.000 | 1.012 | 1.047 | 0.593 | 0.885 | 1.238 | 0.816 | 0.197 | 0.599 | 1.112 | 1.006 | 0.557 | 0.985 | 1.028 | 1.012 | 0.886 | 0.862 | 1.187 |
| **CONGESTION** | 0.997 | 0.469 | 0.990 | 1.005 | 1.126 | 0.203 | 0.938 | 1.353 | 0.570 | **0.002** | 0.400 | 0.812 | 1.014 | 0.112 | 0.997 | 1.030 | 1.064 | 0.500 | 0.889 | 1.274 |
| **HEADACHES** | 1.001 | 0.820 | 0.993 | 1.009 | 1.255 | **0.033** | 1.018 | 1.546 | 0.734 | 0.139 | 0.488 | 1.106 | 1.012 | 0.259 | 0.991 | 1.034 | 1.006 | 0.956 | 0.820 | 1.233 |
| **SORE THROAT** | 0.998 | 0.751 | 0.989 | 1.008 | 0.940 | 0.664 | 0.711 | 1.243 | 0.748 | 0.341 | 0.412 | 1.359 | 1.012 | 0.337 | 0.987 | 1.038 | 1.115 | 0.415 | 0.858 | 1.450 |
| **MYALGIA** | 1.010 | **0.039** | 1.000 | 1.019 | 0.992 | 0.949 | 0.786 | 1.254 | 0.685 | 0.115 | 0.427 | 1.097 | 1.022 | 0.076 | 0.998 | 1.046 | 1.167 | 0.178 | 0.932 | 1.462 |
| **CHILLS** | 1.008 | 0.225 | 0.995 | 1.021 | 0.915 | 0.554 | 0.681 | 1.229 | 0.695 | 0.282 | 0.359 | 1.347 | 1.014 | 0.432 | 0.980 | 1.049 | 0.945 | 0.706 | 0.705 | 1.266 |
| **FEVER** | 1.004 | 0.389 | 0.995 | 1.014 | 0.955 | 0.726 | 0.737 | 1.237 | 0.797 | 0.332 | 0.503 | 1.262 | 1.005 | 0.686 | 0.982 | 1.028 | 0.939 | 0.627 | 0.730 | 1.209 |
| **BREATHLESSNESS** | 1.009 | 0.137 | 0.997 | 1.022 | 1.069 | 0.675 | 0.783 | 1.460 | 0.376 | 0.071 | 0.130 | 1.085 | 1.031 | 0**.012** | 1.007 | 1.056 | 1.449 | 0.019 | 1.064 | 1.973 |
| **LOSS OF APPETITE** | 1.016 | **0.012** | 1.004 | 1.029 | 0.968 | 0.835 | 0.717 | 1.309 | 0.746 | 0.403 | 0.376 | 1.482 | 1.011 | 0.488 | 0.981 | 1.042 | 1.025 | 0.867 | 0.767 | 1.370 |
| **NAUSEA** | 1.008 | 0.110 | 0.998 | 1.018 | 0.953 | 0.762 | 0.699 | 1.299 | 0.759 | 0.416 | 0.391 | 1.475 | 1.009 | 0.541 | 0.981 | 1.037 | 1.019 | 0.898 | 0.762 | 1.364 |
| **DIARRHOEA** | 1.001 | 0.951 | 0.981 | 1.020 | 0.828 | 0.479 | 0.491 | 1.396 | 1.540 | 0.324 | 0.653 | 3.630 | 0.982 | 0.621 | 0.916 | 1.054 | 0.990 | 0.971 | 0.586 | 1.674 |
| **LOSS OF SMELL/TASTE** | 0.998 | 0.747 | 0.985 | 1.011 | 1.093 | 0.527 | 0.830 | 1.440 | 0.734 | 0.453 | 0.328 | 1.646 | 1.010 | 0.521 | 0.980 | 1.041 | 0.975 | 0.858 | 0.741 | 1.284 |

Table 6. The fold-effects of demographics and their 95%CIs on mean number of days of specific symptoms reported during a symptomatic episode restricted to the PCR+ participants. The estimates are the result of fitting a zero-inflated Poisson model on the number of reports within an episode whilst allowing for multiple episodes with events associated with one participant. The analyses also account for the length of the event-episode. The similar analysis including the whole cohort is presented in Table 4 in the main paper.

| **VARIABLE** | **NUMBER OF REPORTS** | | | | | | | | | | | | | | | | | |
| --- | --- | --- | --- | --- | --- | --- | --- | --- | --- | --- | --- | --- | --- | --- | --- | --- | --- | --- |
| **LOSS OF TASTE AND SMELL** | 1 | 2 | 3 | 1 | 2 | 3 | 1 | 2 | 3 | 1 | 2 | 3 | 1 | 2 | 3 | 1 | 2 | 3 |
| **LOSS OF APPETITE** | 1 | 1 | 1 | 2 | 2 | 2 | 3 | 3 | 3 | 1 | 1 | 1 | 1 | 1 | 1 | 3 | 3 | 3 |
| **FEVER** | 1 | 1 | 1 | 1 | 1 | 1 | 1 | 1 | 1 | 1 | 1 | 1 | 2 | 2 | 2 | 3 | 3 | 3 |
| **NOSE CONGESTION** | 1 | 1 | 1 | 1 | 1 | 1 | 1 | 1 | 1 | 1 | 1 | 1 | 1 | 1 | 1 | 1 | 1 | 1 |
| **COUGH** | 1 | 1 | 1 | 1 | 1 | 1 | 1 | 1 | 1 | 2 | 2 | 2 | 1 | 1 | 1 | 3 | 3 | 3 |
| **RUNNY NOSE** | 1 | 1 | 1 | 1 | 1 | 1 | 1 | 1 | 1 | 1 | 1 | 1 | 1 | 1 | 1 | 1 | 1 | 1 |
| **CHILLS** | 1 | 1 | 1 | 1 | 1 | 1 | 1 | 1 | 1 | 1 | 1 | 1 | 1 | 1 | 1 | 1 | 1 | 1 |
| **PROBABILITY OF PCR+** | 0.60 | 0.72 | 0.82 | 0.66 | 0.78 | 0.86 | 0.73 | 0.82 | 0.89 | 0.62 | 0.74 | 0.83 | 0.65 | 0.76 | 0.85 | 0.83 | 0.89 | 0.94 |

Table 8. Examples of various combination of potential bundles of symptoms and their corresponding probabilities of testing positive as predicted by the optimal model above (age is held at 50 years and the ethnicity is assumed White).
